# Avicin D: A Protein Reactive Plant Isoprenoid Dephosphorylates Stat 3 by Regulating Both Kinase and Phosphatase Activities

**DOI:** 10.1371/journal.pone.0005578

**Published:** 2009-05-18

**Authors:** Valsala Haridas, Goshi Nishimura, Zhi-Xiang Xu, Fiona Connolly, Margaret Hanausek, Zbigniew Walaszek, Robert Zoltaszek, Jordan U. Gutterman

**Affiliations:** 1 Department of Systems Biology, University of Texas M. D. Anderson Cancer Center, Houston, Texas, United States of America; 2 Department of Pharmacology, University of Texas Health Science Center, San Antonio, Texas, United States of America; Bauer Research Foundation, United States of America

## Abstract

Avicins, a class of electrophilic triterpenoids with pro-apoptotic, anti-inflammatory and antioxidant properties, have been shown to induce redox-dependant post-translational modification of cysteine residues to regulate protein function. Based on (a) the cross-talk that occurs between redox and phosphorylation processes, and (b) the role of Stat3 in the process of apoptosis and carcinogenesis, we chose to study the effects of avicins on the processes of phosphorylation/dephosphorylation in Stat3. Avicins dephosphorylate Stat3 in a variety of human tumor cell lines, leading to a decrease in the transcriptional activity of Stat3. The expression of Stat3-regulated proteins such as c-myc, cyclin D1, Bcl2, survivin and VEGF were reduced in response to avicin treatment. Underlying avicin-induced dephosphorylation of Stat3 was dephosphorylation of JAKs, as well as activation of protein phosphatase-1. Downregulation of both Stat3 activity and expression of Stat 3-controlled pro-survival proteins, contributes to the induction of apoptosis in avicin treated tumor cells. Based on the role of Stat3 in inflammation and wounding, and the *in vivo* inhibition of VEGF by avicins in a mouse skin carcinogenesis model, it is likely that avicin-induced inhibition of Stat3 activity results in the suppression of the pro-inflammatory and pro-oxidant stromal environment of tumors. Activation of PP-1, which also acts as a cellular economizer, combined with the redox regulation by avicins, can aid in redirecting metabolism from growth promoting anabolic to energy sparing pathways.

## Introduction

Coincident with the identification of the human genome, as well as increased understanding of gene networks, a quiet renaissance is occurring in the development of natural products as drug candidates [1]. Although almost one-half of all current medications are plant products or their derivatives, the often superior specificity and potency of these small molecules (<2500 Da) compared to synthetic molecules is not widely appreciated. Their size, three dimensional structure, as well as functionality often make natural products outstanding drug candidates or pharmacophores for novel and imaginative new drugs [2]. The interest in natural products parallels the important advances in natural product chemistry, bioengineering, and biocatalysis [3].

Avicins, a family of desert plant-derived triterpenes that have been identified, purified and characterized by our group, offers interesting possibilities for treating complex diseases of ageing, where targeting single proteins may not offer optimal results. The extraction and purification of avicins from the ground pods of Acacia victoriae have been described in detail by Jayatilake et al [4]. Using induction of cell cytotoxicity as a screen, two fractions namely avicin D and avicin G were identified as most active [4]–[6]. The HPLC profile of the material used and the chemical strucures of the two avicins have been shown in Supplemental information (Figure S1). The avicin D obtained as a white amorphous powder is about 96% pure and has a molecular weight of 2,104 atomic mass units [4]. Further details regarding the chemistry of the avicin molecules have been described earlier by [4]. Based on the higher recovery of avicin D fraction, we chose to use it for all subsequent studies. Avicin D has been shown to inhibit NF-κB [7] and activate NF-E2-related factor 2 (Nrf2) [8] respectively, both in a redox-dependant manner, accounting for its anti-inflammatory [7], [9] and antioxidant properties [8]. The ability of avicins to interact with, and modify cysteine residues was first demonstrated in a bacterial system with OxyR as a target, wherein we demonstrated that the distal portion of the avicin side chain formed a reversible and covalent thioester bond with the critical cysteine (SH) on the OxyR molecule [10]. This protein modification, termed avicinylation, suggested to us that avicins can induce post-translational changes in proteins to regulate their function.

Post-translational modifications of proteins are important ways by which small molecules modulate human physiology [11]. Often different post-translational modifications occur in tandem, and the cross talk between these modifications determines the final cellular outcome. One such protein modification that often accompanies thiol modifications is the phosphorylation/dephosphorylation of proteins. We have demonstrated that avicins inhibit oxygen consumption and ATP generation in healthy as well as tumor cells [12]–[14]. Decrease in the generation of cellular ATP has an inhibitory effect on protein-phosphorylation [15], thereby significantly affecting various signaling pathways [16]. In order to study the role of avicins in regulating the phosphorylation and thereby function of proteins, we chose to study the effects of avicin D on Stat 3 signaling. Stat3, a regulator of cell survival, wounding and metabolism is constitutively activated by phosphorylation in most cancers [17], [18]. Targeting the Stat signaling pathway could provide an important mode of anti-cancer therapy [17], based on role of Stat proteins in regulation of cell proliferation, differentiation, survival, malignant transformation and oncogenesis [18].

## Results

### Avicin D inhibits constitutive and induced Stat3 phosphorylation in multiple myeloma cells

**Figure 1:**
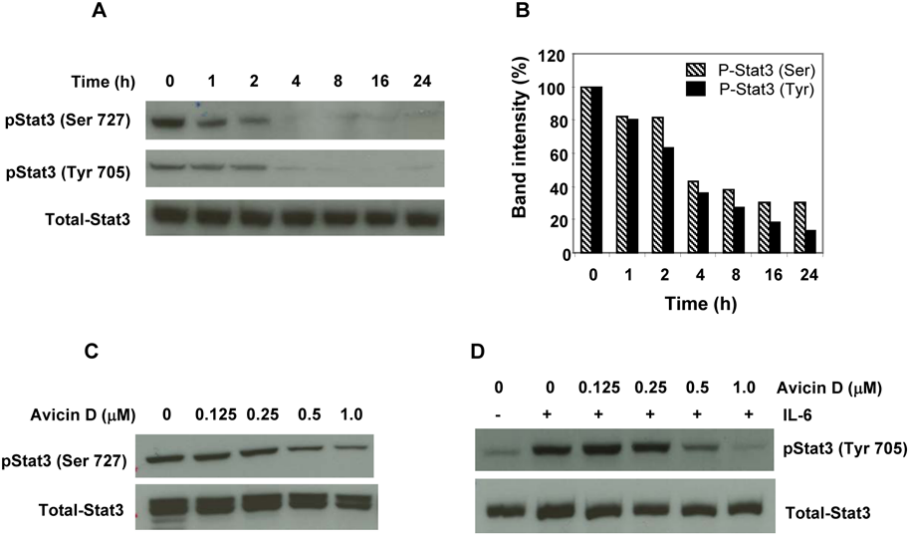
Avicin D dephosphorylates Stat3 both in a time and dose-dependant manner. (A) U266 cells were treated with 1 µM of avicin D for 0–24 hrs. Whole cell lysates were analyzed for phospho-Stat3 (Ser 727 and Tyr 705) and total Stat3 expression by Western blot analysis as described in the methods. (B) Quantitation of the total and phospho-Stat3 bands from Fig. 1A. (C) U266 cells were treated with 0–1 µM of avicin D for 16 hrs. Whole cells lysates were analyzed for phospho-Stat3 (Ser 727) levels. (D) U266 cells pre-treated with 0–1 µM of avicin D (16 h) were then exposed to IL-6 (10 ng/ml) for 30 min. Whole cell lysates were analyzed for phospho-Stat3 (Tyr 705) levels by western blot analysis.

One of the critical steps leading to the activation of Stat3 is its phosphorylation at Tyr 705 and/or Ser727. Treatment of U266 cells with avicin D (1 µM), resulted in a time-dependant decrease in Stat3 phosphorylation, both at Tyr 705 and Ser 727, with no significant changes in the levels of total Stat3 (Fig. 1A). A 20% reduction in levels of phospho-Stat3 (Ser 727 and Tyr 705) was observed within 1 hr of treatment, and at the end of 24 hr only about 15–35% of the phospho- Stat3 was detectable (Fig. 1B).

**Figure 2:**
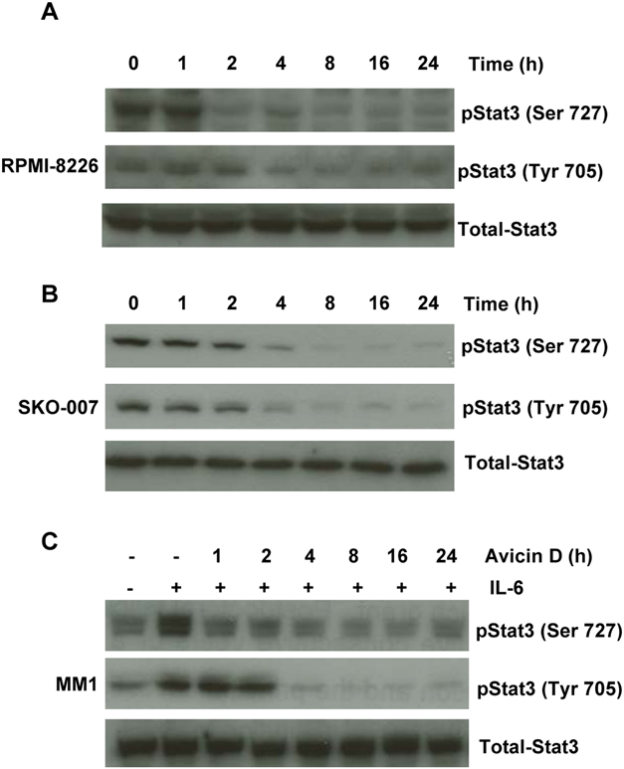
Avicin D dephosphorylates both constitutive and induced Stat3 in myeloma cell lines. (A) RPMI-8226, (B) SKO-007 and (C) MM1 cells were treated with 1 µM of avicin D for 0–24 hrs. At the end of the avicin D treatment, MM1 cells were additionally exposed to IL-6 (10 ng/ml, 30 min). Whole cell lysates were analyzed for phospho-Stat3 (Ser 727 and Tyr 705) and total Stat3 expression by Western blot analysis as described in the methods.

We next evaluated the optimal dose of avicin D required for dephosphorylation of both constitutive and IL-6 induced Stat3 in U266 cells. As shown in Fig. 1C 0.5–1.0 µM of avicin D (16 hr) was required to dephosphorylate Stat3 at Ser 727, while lower doses had no effect. Dephosphorylation of IL-6 induced phospho-Stat3 (Tyr 705) also required at least 0.5 µM of avicin D (Fig 2D). In all the above studies, no significant changes were observed in the levels of total Stat3. Based on these results, for the rest of the study, we chose to treat cells with 1 µM of avicin D for 16 hr.

To demonstrate that the avicin-mediated regulation of Stat3 was not restricted to U266 cells, we studied some other multiple myeloma cell lines. These cells have been found to be sensitive to avicin-induced killing [19]. A time dependant decrease in the constitutive levels of phospho-Stat3 (Ser 727 and Tyr 705) was observed in RPMI-8226 and SKO-007 cells (Fig. 2A&B). In MM1 cells, IL-6 induced phosphorylation of Stat3 was inhibited in response to avicin D in a time dependant manner (Fig. 2C).

### Avicin D inhibits the nuclear localization of Stat3 in myeloma cells

**Figure 3:**
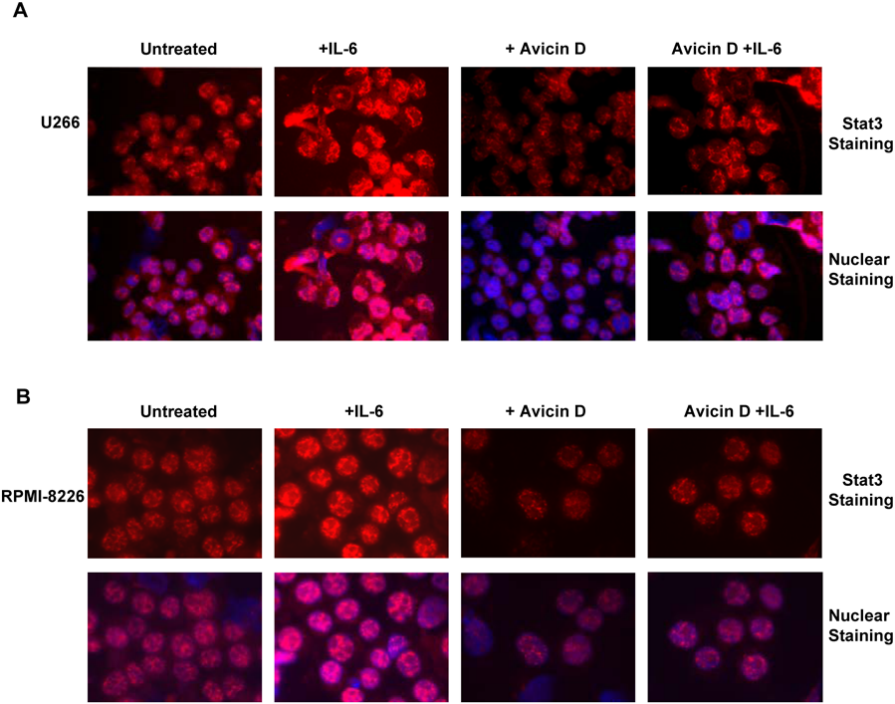
Cellular localization of Stat3 in avicin D-treated myeloma cells. U266 (A) and RPMI-8226 (B) cells pre-treated with 1 µM of avicin D for 16 hrs were exposed to IL-6 (10 ng/ml) for 30 min. Staining and immunohistochemistry of Stat3 was done as described in the methods.

In the absence of cytokine stimulation, Stats exist as stable, non-phosphorylated dimers, which constantly shuttle between the cytoplasm and nucleus [20]. However, the phosphorylated Stat protein is retained in the nucleus and is only released upon dephosphorylation by nuclear phosphatases. Therefore, besides nuclear translocation, nuclear retention of phosphorylated Stat is also crucial for its activation. We studied the effect of avicin D on the localization of both constitutive and IL-6 induced Stat3 in U266 (Fig. 3A) and RPMI-8226 (Fig. 3B) cells. Consistent with avicin-induced dephosphorylation of Stat3, is the observation that following avicin D treatment, both untreated and IL-6 treated cells show reduced presence of Stat3 in the nucleus. This could be due inhibition in the nuclear translocation and/or nuclear retention of Stat3. These results suggested to us that avicin D by dephosphorylating Stat3 and inhibiting its nuclear localization, could affect the transcriptional activity of Stat3.

### Avicin D inhibits Stat3-dependant gene expression

**Figure 4:**
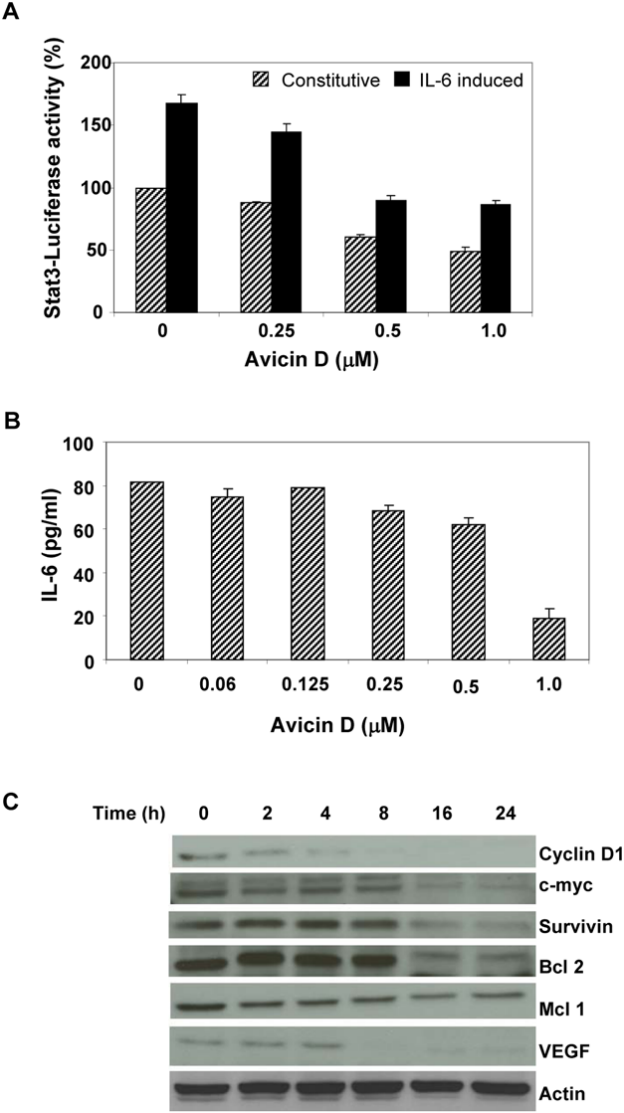
Avicin D downregulates Stat3-induced protein expression. (A) Effect of avicin D on Stat3 -mediated luciferase gene expression. U266 cells were transfected with Ly6E-Luciferase-Stat3 plasmid as described in the methods. 24 hrs later cells were washed and treated first with avicin D (1 µM) for 16 hrs, and then exposed to IL-6 (10 ng/ml) for 30 min. Luciferase activity was measured as described in the methods. The luciferase activity of the treated samples is expressed relative to the activity of untreated control (no avicin, no IL-6), which is taken as 100%. (B) Effect of avicin D on IL-6 production in U266 cells. Cells were treated with 0–1 µM of avicin D for 48 hrs. Cell supernatants were assayed for IL-6 levels using an ELISA kit. (C) Effect of avicin D on the expression of Stat3 regulated proteins. U266 cells were treated with 1 µM of avicin D for 0–24 hrs. Whole cell lysates were analyzed for the expression of different proteins by western blot analysis as described in the methods.

Phosphorylated Stat3 in the nucleus binds to DNA and regulates the transcription of several target genes. U266 cells transiently transfected with Ly6E Stat3-luciferase construct were treated with avicin D. Constitutive, and IL-6 induced Stat3 activity was assessed by measuring the luciferase activity. Pre-treatment of cells with avicin D resulted in a significant decrease in both the constitutive as well as IL-6 induced Stat3-luciferase activity (Fig. 4A), suggesting a decrease in the transcriptional potential of Stat3.

IL-6, a major activator of Stat3, particularly in myeloma cells, is known to be under the transcriptional regulation of Stat3 itself, thereby constituting a feedback loop [21], [22]. To study the effect of avicin D on Stat3-induced IL-6 production, we assayed the cell culture medium for IL-6 levels. U266 cells treated for 48 hrs with avicin D, showed a dose dependant decrease in IL-6 production (Fig. 4B), which is consistent with the avicin-induced inhibition of Stat3 phosphorylation and transcriptional activity.

To further demonstrate the reduced transcriptional activity of Stat3 in avicin-treated cells, we studied the expression of Stat3 regulated proteins such as cyclin D1, c-myc, survivin, Bcl-2, Mcl-1, and VEGF. As shown in Fig. 4C, U266 cells treated with avicin D showed a time-dependant decrease in levels of these proteins. While levels of cyclin-D, Mcl-1 and VEGF began to decline within 2–4 hrs of avicin D treatment, Bcl2 and survivin levels decreased following longer (8–16 hrs) treatments. These findings are in confirmation with an earlier study, showing that avicin D selectively induces apoptosis and down regulates pro-apoptotic proteins in cutaneous T-cell lymphoma cells [23].

### Role of kinases in avicin D-induced dephosphorylation of Stat3

**Figure 5:**
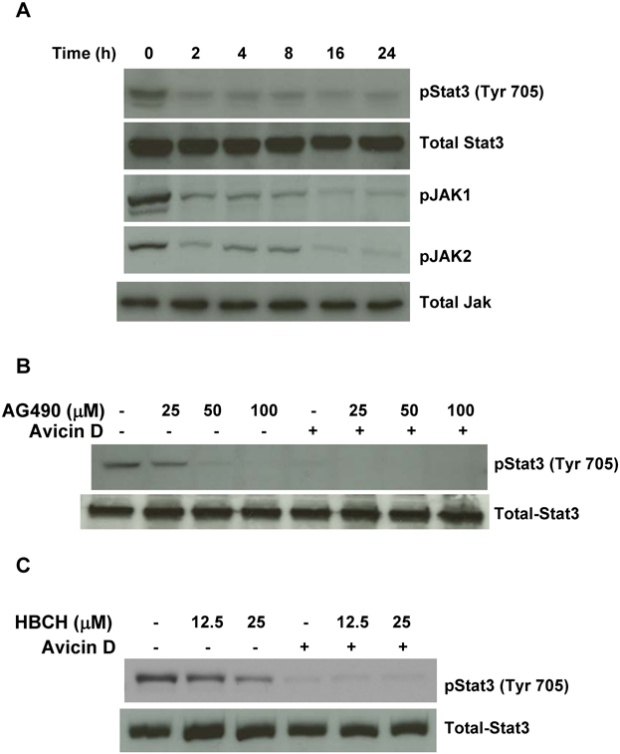
Effect of avicin D on Janus kinases in U266 cells. (A) U266 cells were treated with avicin D (1 µM) for 0–24 h. Whole cell lysates were assayed for expression of phospho-JAK1, phospho-JAK2 and total JAK, using western blot analysis. These results were analyzed against changes in the levels of phospho-Stat3 (Tyr 705). (B) Effect of AG490 on avicin D-induced dephosphorylation of Stat3 (Tyr 705). U266 cells were pretreated with AG490 (0–100 µM) for 2 hrs, followed by a 16 hrs exposure to avicin D (1 µM). Cells treated with AG490 alone, were treated for 18 h with the inhibitor. Whole cell lysates were assayed for expression of phospho-Stat3 and total Stat3 using western blot analysis. (C) Effect of HCBH on avicin D-induced dephosphorylation of Stat3 (Tyr 705). U266 cells were pretreated with HCBH (0–25 µM) for 2 hrs, followed by a 16 hrs exposure to avicin D (1 µM). Cells treated with HCBH alone, were treated for 18 h with the inhibitor. Whole cell lysates were assayed for expression of phospho-Stat3 and total Stat3 using western blot analysis.

Based on the observations so far, we were interested in understanding the mechanism(s) underlying avicin D-induced dephosphorylation of Stat3. Dephosphorylation of a protein can be a result of either inactivation of specific kinases or activation of phosphatases. Janus kinases (JAKs) upon phosphorylation are known to activate Stat3 in response to various stimuli such as cytokines, and growth factors [24], [25]. Treatment of U266 cells with avicin D for 0–24 h resulted in dephosphorylation of both JAK1 and JAK2, concomitant with the dephosphorylation of Stat3 at Tyr 705 (Fig. 5A). No change in total JAK levels was observed. Of the different members of the JAK family, JAK2 is known to be involved in the tyrosine phosphorylation of Stat3 [26]. Based on the finding that avicin D inhibits JAK2, we next wanted to see how avicin D compares with known inhibitors of JAK2. For this purpose, we used tyrphostin (AG490) and hexabromocyclohexane (HBCH). While HBCH is a specific inhibitor of JAK2 [27], AG490 targets both JAK2 and JAK3 [28]. Almost total dephosphorylation of Stat3 was achieved with 1 µM of avicin D, whereas much higher doses of AG490 (50–100 µM) (Fig 5B) and HCBH (more than 25 µM) (Fig. 5C) were required for a comparable effect. At the concentrations studied, avicin D in combination with either of the inhibitors was as effective as by itself in dephosphorylating Stat3 (Figs 5B & C). These findings demonstrate the better potency of avicin D compared to some of the known JAK2 inhibitors.

### Role of phosphatases in avicin D-induced dephosphorylation of Stat3

**Figure 6:**
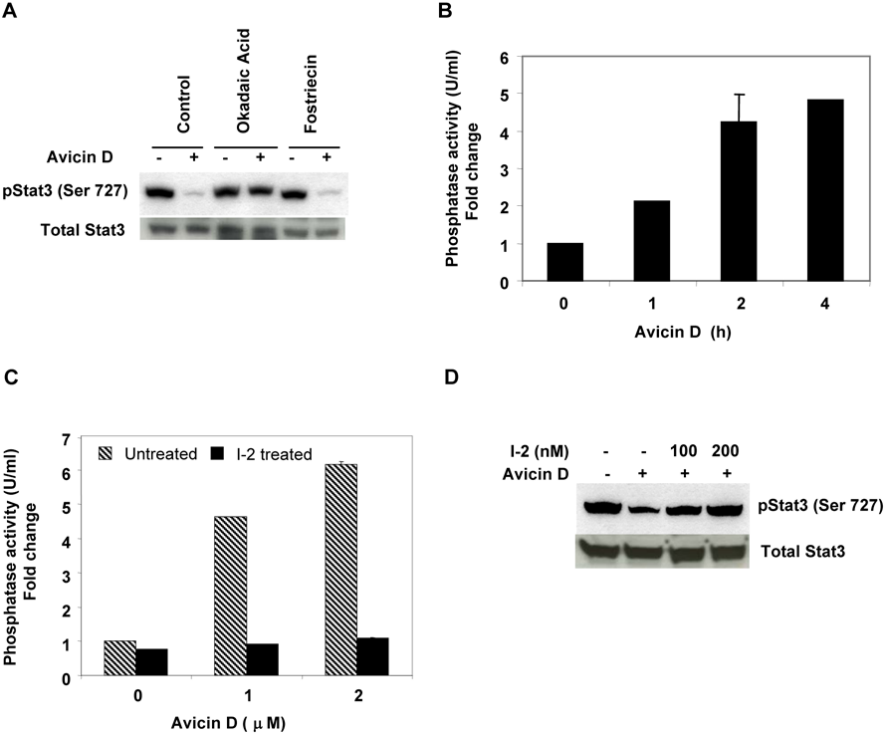
Avicins activate a ser/Thr phosphatase. (A) Effect of phosphatase inhibitors on avicin D-induced dephosphorylation of Stat3. Cells were treated with okadaic acid (1 µM) or fostriecin (1 µM) for 1 hr prior to a 4 hr treatment with avicin D (1 µM). Whole cell lysates were analyzed for phospho-Stat3 (ser 727) and total Stat3 expression by Western blot analysis as described in the methods. (B) Avicin D enhances PP1 activity in U266 cells. Cells were treated with 1 µM of avicin D for 0–4 hrs. PP1 was immunoprecipitated and its activity was assayed as described in the methods. (C) Avicin D-induced PP1 activity is blocked by I-2. PP1 was immunoprecipated from avicin D treated (1 µM, 4 hrs) U266 cell lysates. 200 nM of I-2 was added into the reaction mix and phosphatase activity was measured as described in the methods. (D) Avicin D-induced Stat3 dephosphorylation is reversed by I-2. U266 cells were treated with 100- and 200 nM of I-2 for 20 hrs prior to a 4 hr treatment with avicin D (1 µM). Whole cell lysates were analyzed for phospho-Stat3 (ser 727) and total Stat3 expression by Western blot analysis as described in the methods.

While the tyrosine phosphorylation of Stat3 and the kinases involved have been extensively studied, very little is known about the mechanisms underlying serine phosphorylation of Stat3. Some of the kinases which have been suggested to play a role in serine phosphorylation of Stat3 (MAPK, PI3K, PKCδ and JNK) are all either activated in response to avicin D, or are inhibited at a much later time point. In the absence of any clear indications that avicins inactivate a serine kinase to dephosphorylate Stat3, we decided to evaluate the involved phosphatases. We first studied the effect of phosphatase inhibitors on avicin-induced dephosphorylation of Stat3 in U266 cells. Okadaic acid (OA), an inhibitor of PP1/PP2A completely reversed the avicin-induced dephosphorylation, whereas fostriecin, a PP2A/PP4 inhibitor had no effect at all (Fig. 6A), suggesting the probable involvement of PP1. We next measured the phosphatase activity in avicin D-treated U266 cells using P^32^ labeled myelin basic protein as a substrate. As shown in Fig. 6B, a 5 fold increase in PP1 activity was observed following a 4 h treatment with avicin D. To further confirm the role of PP1 in avicin-induced Stat3 dephosphorylation, we used inhibitor-2 (I-2) a specific PP1 inhibitor. In an *in vitro* assay, I-2 totally inhibited the phosphatase activity in the lysates of avicin D-treated U266 cells (Fig. 6C). Pretreatment of U266 cells with I-2 for 20 h, significantly blocked avicin-mediated dephosphorylation of Stat3 (Fig. 6D), strongly suggesting the involvement of PP1. All these results clearly point to the role of PP1 in avicin-mediated serine dephosphorylation of Stat3.

### Down regulation of Stat3 is involved in avicin-induced cell death

**Figure 7:**
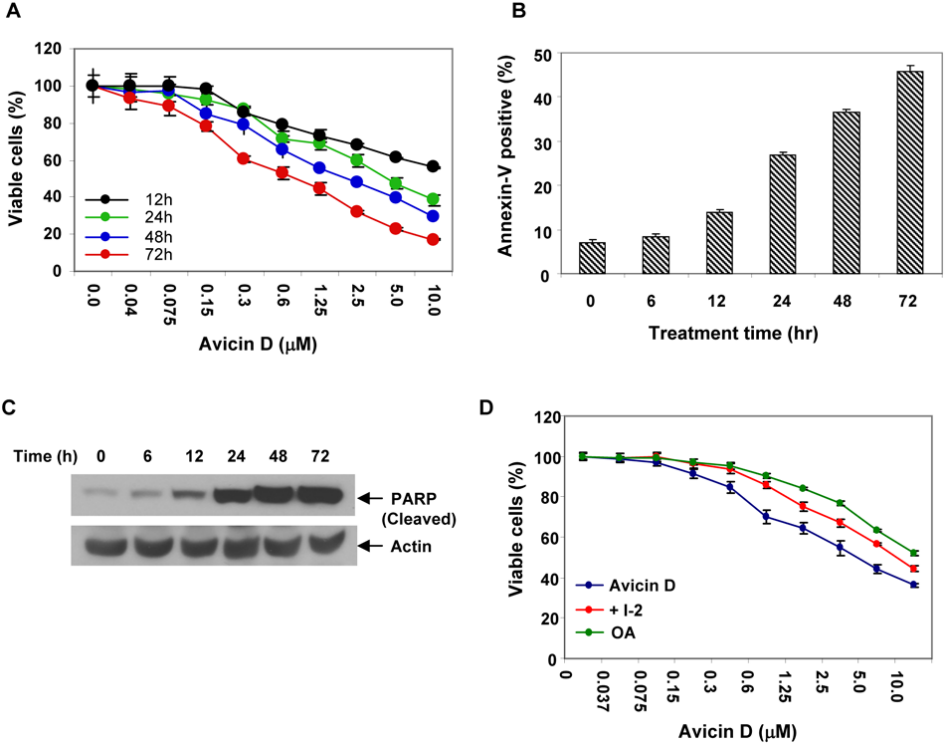
Avicin D induced dephosphorylation and apoptosis are related. (A) The growth inhibitory activity avicin D was measured by the MTT assay. Cells (1×10^4^/well) were cultured with 0–10 µM of avicin D in 96-well plates for 0–72 h at 37°C. MTT assay was performed as described in the methods. (B) Annexin V-FITC binding in avicin D-treated U266 cells. U266 cells (1×10^6^/ml) were treated with 1 µM of avicin D for 0–72 hrs. Cells were stained and analyzed by flow cytometry as described in the methods. (C) Cleavage of PARP by avicin D. U266 cells (1×10^6^) were treated with 1 µM of avicin D for 0–72 hrs. Whole cell lysates were prepared and assayed for expression of cleaved PARP, using western blot analysis as described in the methods. (D) Effect of OA and I-2 on avicin-induced cell death. In a 96-well plate U266 cells (1×10^4^/well) were pretreated with OA (100 nM) and I-2 (100 nM) for 12 hr. Next the avicin D (0–10 µM) was added to the culture and incubated for 24 h at 37°C. MTT assay was performed as described in the methods.

Avicin D has been shown to induce cell death in a variety of human tumor cells [6], including multiple myelomas [19]. The findings reported here, suggest that targeting the Stat3 pathway could be a possible mechanism underlying avicin-induced cell death. To investigate this, we first evaluated the sensitivity of U266 cells to avicin-induced cell death. Avicin D treated U266 cells showed a dose and time-dependant inhibition of viability (Fig. 7A). Any inference by avicin D in the assay due to its interaction with MTT was ruled out as described in the methods.

To understand the mechanism of growth inhibition, U266 cells treated with avicin D for 0–72 hrs were analyzed for annexin V-FITC binding. Cells were simultaneously stained with propidium iodide to check for their viability. Shown in Figure 7B, is a time dependant increase in annexin V positive cells, indicative of apoptosis. To further confirm the induction of apoptosis we assayed avicin D-treated U266 cell lysates for cleavage of PARP, a hallmark of apoptosis. Avicin D induced cleavage of PARP was clearly detectable at 12 hr of treatment (Fig 7C), which is consistent with the annexin V data.

After establishing that avicin D induces apoptosis in U266 cells, we next asked if the apoptosis was related to the dephosphorylation of Stat3. To address this question, we studied the effect of OA and I-2, PP1 inhibitors which have been earlier shown to reverse avicin D-induced dephosphorylation of Stat3, on the induction of cell death. As shown in Fig. 7D, U266 cells pre-treated with OA and I-2, showed a partial inhibition of avicin D-induced cell death. The IC 50 (concentration required to induce 50% cell kill) value for avicin D alone was 1.4 µM, while in the presence of OA and I-2, this value rose to 5.75 and 10.4 µM respectively. These findings clearly indicate that inhibition of Stat3 does play a role in the mediation of cell death following avicin treatment. The lack of complete inhibition of avicin D-induced cell death in this experiment can be explained by the fact that (a) only a part of the Stat3 dephosphorylation (at Ser 727) was reversed and (b) there are other pathways shown to be involved in avicin-induced cell death [5]–[7], [29], which are not targeted by OA and I-2.

### Avicin D induces dephosphorylation of Stat3 in a wide variety of human tumors

**Figure 8:**
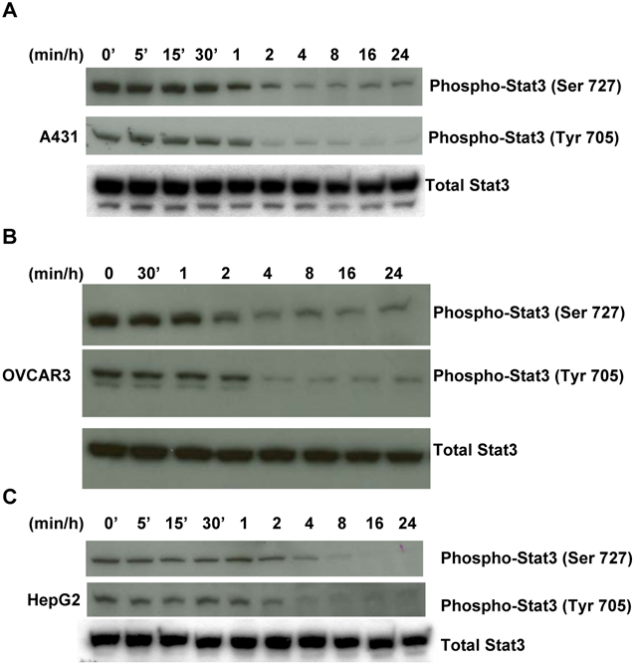
Avicin D dephosphorylates Stat3 in a variety of human tumor cell lines. Cells were treated with 1 µM of avicin D for 0–24 hrs. Whole cell lysates were analyzed for phospho-Stat3 and total Stat3 by Western blot analysis as described in the methods.

Since Stat3 is constitutively activated in various human tumors besides myelomas, we questioned if avicin D would regulate the Stat3 activity in these tumors too. We selected three human cell lines of different origins that have been found to be sensitive to avicin-induced killing. Shown in Fig. 8 is the dephosphorylation of Stat3 in A431 (epidermoid), OVCAR3 (ovarian) and HepG2 (hepatocarcinoma) cells treated with avicin D. These results indicate that avicins target the Stat3 pathway in a wide variety of human tumors.

### Avicin D lowers the levels of VEGF and CD31 in mouse skin

To extrapolate the above-described effects of avicins to an *in vivo* system, we used a mouse skin carcinogenesis model. Based on recent reports [30] suggesting the role of Stat3 in the expression of VEGF and tumor angiogenesis, we evaluated the levels of VEGF in the skin sections of mice treated with DMBA. SENCAR mice skin was treated with either acetone (control) or avicin D as described in the methods, prior to treatment with DMBA.

The mean weights of the animals in all treatment groups were analyzed by one-way ANNOVA. There were no significant differences in body weight, body weight gains or food consumption between animals treated with acetone or avicins, suggesting no dramatic changes in the performance of treated animals. Tumor progression was monitored by measuring epidermal hyperplasia and total dermal cellularity as described earlier [9]. The histological evaluation of tumor progression and effect of avicins on it have also been described previously [9].

**Figure 9:**
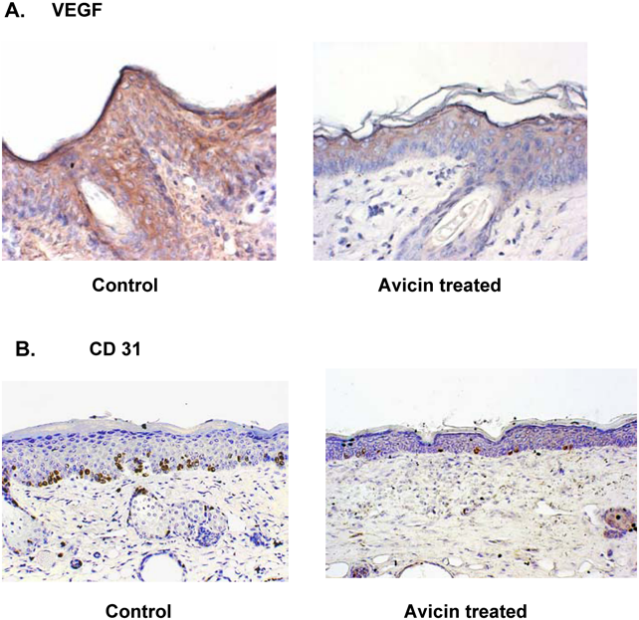
Effect of avicin D on levels of VEGF and CD31 in mouse skin. Mice treated with acetone (control) or avicin were exposed to DMBA for 8 weeks. At the end of the treatment, skin sections were analyzed for (A) VEGF and (B) CD 31 levels as described in the methods (H&E Staining; ×400).

Immunohistochemistry studies revealed that avicin treated skin showed a dramatic decrease in the levels of VEGF, as compared to the acetone treated (control) skin (Fig. 9A). To ensure that the decreased levels of VEGF translated into decrease in angiogenesis, we next studied the levels of CD31, an endothelial cell marker that is indicative of the microvascular networks within the cells. As shown in Fig. 9B, avicin treatment induced a decrease in the CD31 levels, as compared to the control demonstrating the anti-angiogenic potential of avicins.

## Discussion

Post-translational protein modification is emerging as an important regulatory control over cellular function [11]. Here we demonstrate avicin D-induced dephosphorylation of Stat3, and down regulation of Stat3-dependant proteins. Stat3, persistently activated in several types of human cancers has been now shown to play a critical role in the process of carcinogenesis [31]–[33] by enhancing tumor cell proliferation, survival as well as cellular transformation [33], [34]. Based on its involvement in the process and maintenance of oncogenesis, activity of Stat3 as a transcriptional activator is a prime target for anticancer interventions.

Avicins a family of plant stress metabolites have been shown to induce more than one form of posttranslational modification in different proteins [10], [35], which in turn translates into some of its key properties. Avicins were first characterized as molecules that specifically induce apoptosis by direct perturbation of the mitochondria [5], in a wide variety of human tumors. NF-κB and Nrf2, both transcription factors containing cysteine residues critical for their activity, are regulated by avicins in a redox sensitive manner [7], [8], probably accounting for avicins' anti-inflammatory and cytoprotective properties. Avicins have also been shown to reduce oxygen consumption and levels of cellular ATP [13], [14], which can lead to a hypo metabolic state [12]–[14]. The lowering of cellular ATP levels could contribute to avicins' ability to dephosphorylate proteins and thereby regulate their function [6]. In the present study we focused on the effects of avicins on Stat3 phosphorylation. Avicin D treatment resulted in dephosphorylation of both constitutive and induced Stat3 in a time and dose-dependant manner, leading to a decrease in the transcriptional activity of Stat3. Cultures of avicin treated myeloma cells showed a decrease in the levels of IL-6, a major inducer of Stat3 activity, which is also known to be under the transcriptional regulation of Stat3 [21], [22]. This decrease in IL-6 levels could also contribute to the decrease in Stat3 activation, in avicin treated cells. In addition to IL-6, the expression of cyclin D1, c-myc, survivin, Bcl2, Mcl1, and VEGF, all of which are regulated by Stat3 activity, and play a crucial role in apoptosis and progression of cancer [33], are also down regulated in response to avicin D.

Tyrosine phosphorylation of Stats has been extensively studied, and the role of JAKs is well accepted [24]–[26]. Dephosphorylation of JAK2 appears to be a mechanism for avicin D-induced dehosphorylation of Stat3 at Tyr 705, though the involvement of other members of the JAK family such as JAK1 or JAK3 cannot be ruled out. Serine phosphorylation of Stats is much less understood, and the involved kinases are not clearly known. Recent reports suggest the involvement of PI3K, PKC δ, ERK, JNK and mTOR in serine phosphorylation of Stat3 [26]–[40]. Avicins have been shown to inhibit PI3K [6], mTOR [29], and PKC δ (unpublished data), though long (12–16 hrs of avicin treatment) after Stat3 dephosphorylation is observed. ERK and JNK are both activated early on in avicin treated cells (unpublished results). There are some studies suggesting that activation of MEKs and ERKs can antagonize, and thereby inhibit IL-6 induced JAK-Stat signaling [41]. However, PD98059, an inhibitor of MEK1/2, had no significant effects on avicin-induced dephosphorylation of Stat3 (Ser 727) (data not shown), thereby ruling out the possible involvement of ERK activation in avicin-induced Stat3 (Ser 727) dephosphorylation. In the absence of any clear indications that avicins inactivate a serine kinase, we next asked whether any ser/Thr phosphatase activity was enhanced. . Majority of the known serine/threonine phosphatases belong to the PP1 and PP2A families. The net hydrolysis of phosphoserine (pS) and phosphotyrosine (pT) versus phosphotyrosine (pY) residues is believed to be mediated via distinct yet related mechanisms [10]. One study using phosphatase inhibitors have implicated the role of PP2A in the dephosphorylation of Stat3 [42]. While fostriecin a PP2A/PP4 inhibitor had no effect on avicin-induced dephosphorylation of Stat3, OA and inhibitor-2 (I-2), both PP1 inhibitors, completely reversed avicin D-induced dephosphorylation of Stat 3 (Ser 727), suggesting the probable involvement of PP1. Using an *in vitro* serine/threonine phosphatase assay system, we found that avicin D induces PP1 activity. Taken together, these results suggest a role for PP1 in Stat3 dephosphorylation by avicins, though an additional involvement of PP2A cannot be completely ruled out.

The activation of a phosphatase that can deactivate phosphorylation (serine) of an important oncogenic signaling molecule, reported in this paper is a novel finding. How exactly the avicin molecule activates PP1 is not clear. The ability of the avicin molecule to bind to SH groups could regulate the activation of Fe and/or Zn in the bimetallic center. Alternatively, avicins might inhibit the endogeneous inhibitors of PP1 (DARPP32, I-1 and I-2), which also appear to affect the B12–B13 loop [43].

Stat3 as mentioned earlier is activated in several human tumors, rendering them resistant to apoptotsis by regulating various anti-apoptotic proteins [31]. Avicin D-induced dephosphorylation of Stat3 and the subsequent inhibition of its transcriptional activity, contributes to sensitizing the cells to apoptosis. This was shown in U266 cells, which undergo apoptotsis in response to avicin treatment. Upon inhibition of Stat3 dephosphorylation (Ser 727) by using PP1 inhibitors, avicin D-induced cell death was inhibited. Hence targeting Stat3 could be another mechanism by which avicins induce cell death. The ability of avicins to target degenerate pathways leading to cell death, in a systems manner is likely to make it more effective in a variety of tumor cells which might be mutated for one or the other pathways.

Studies from DiGiovanni's laboratory have shown the involvement of Stat3 in both the initiation and promotion of epithelial carcinogenesis [44]. One of the key requirements for successful tumorigenesis is the establishment of neovascularization or angiogenesis, in order to acquire nutrients for continued growth and metastatic spread [45]. VEGF, a key inducer of angiogenesis [46], is believed to be regulated mainly by two transcription activators, namely hypoxia inducible factor-1 (HIF-1) and Stat3 [47]. Recent studies have shown that Stat3 regulates the basal as well as growth signal-induced expression of HIF-1 [47], which like Stat3 is up regulated in a variety of human tumors. HIF-1 in turn mediates PI3K/Akt-induced VEGF expression [48]. Unpublished results from our laboratory also show avicins down regulate HIF-1 levels in human tumor cells. Skin sections from avicin-treated mice showed dramatic decrease in the levels of VEGF accompanied by a decrease in CD31 staining, which is indicative of a reduction in angiogenesis. Avicins therefore can inhibit VEGF expression by down regulating both the Stat3 as well as HIF-1 activity.

One of the underlying causes of carcinogenesis is believed to be sustained cellular stress, and chronic cellular injury leading to wounds that fail to heal causing unabated inflammation [49], [50]. Thus, chronic exposure to UV light, chemical carcinogens contained in tobacco products, as well as environmental toxins and saturated fats, often result in tissue injury, inflammation, insufficient repair, exposure to oxygen and nitrogen radicals and eventually in the selection of mutations and genomic instability. Tumors characterized by inflammatory or wounding phenotype, have been found to respond poorly to treatment, as compared to tumors that do not show this phenotype [50]. Stat3 forms a nodal point of an assembly of proteins that regulate wounding, chronic injury, inflammation, and tumor cell survival [51]. Recent studies have implicated the role of Stat3 in inducing a subset of pro-inflammatory IL-17-producing helper T cells (THi) [52] Constitutive activation of Stat3 has been found in intestinal T cells from Crohn's disease patients [53]. Constitutive activation of Stat3 is also observed in various tumors and is correlated with poor prognosis [54]–[58]. Considering the pivotal role Stat3 plays in these processes, the ability of avicins to suppress Stat3 signaling and transcriptional activity supports the idea of avicin, or an analog, as a candidate drug for malignancy. This idea is further supported by the exquisite *in vitro* sensitivity of multiple myeloma cells, which express high levels of constitutively activated Stat3, to avicins [19].

Many signals and signaling systems are shared between plants and mammals [59]. So it now should be no surprise to discover a plant molecule to which human cells can sense and respond to. For example, other plant compounds such as epigallocatechin-3-gallate (EGCG) and curcumin have also been shown to regulate phosphorylation and activity of Stat3 in human cells [60], [61]. A new field of chemical biology, which studies the metabolome of lower organisms and how these small molecules perturb human biological targets, is emerging [16]. These so-called secondary metabolites, which are used by plants as defensive molecules, clearly can influence many biological functions in human cells such as immunity, behavior, reproduction, and growth and are serving as new leads for pharmaceuticals.

The emerging recognition of the intersection of metabolic stress, inflammation, wounding with diseases of aging such as cancer has stimulated a search for compounds that can re-establish metabolic homeostasis. Stat3 sits at the nexus point in signaling pathways and transcription in these processes. The ability of avicins, a natural product shaped by a million years of evolutionary selection, to regulate post-translational protein changes, in particular redox and phosphorylation/dephosphorylation, offers new insights in the potential treatment of metabolic stress. Protein-reactive natural products, in particular electrophiles, have become an important focal point for the discovery of new drug candidates [62]. The ability to activate PP1, a cellular economizer [63], together with the above mentioned effects of avicins on cellular metabolism, suggest that further studies of the chemical biology of this compound should yield important insights for therapeutic intervention.

## Materials and Methods

### Cell lines

All the cell lines used were of human origin. U266, RPMI-8226, SKO-007 and MM1 (all multiple myelomas), A431 (epidermoid), and OVCAR3 (ovarian), were maintained in RPMI 1640. HepG2 (hepatoblastoma) cells were grown in alpha-MEM. All the media were supplemented with 10% FBS and 2 mM l-glutamine.

### Reagents and kits

Avicins were extracted and purified from ground seed pods of Acacia victoriae as described earlier [4]. Anti-Stat3, -phospho Stat3 (Ser 727), -phospho Stat3 (Tyr 705), -cleaved PARP, -cyclin D1, -c-myc, -Bcl-2 and -Bcl-xL antibodies were all purchased from Cell Signaling Technology (Beverly, MA ). Anti- survivin, anti-VEGF and anti-CD31 antibodies were obtained from Novus Biologicals (Littleton, CO), Abcam Inc., (Cambridge, MA) and BD Biosciences (San Jose, CA) respectively. Anti-PP-1 antibody was purchased from Santa Cruz Biotechnology (Santa Cruz, CA). AG 490 and Hexabromocyclohexane (HBCH) were purchased from Calbiochem (Gibbstown, NJ). Human interleukin-6 (IL-6) and hu IL-6 ELISA kit were purchased from BD Pharmingen (San Jose, CA) and R&D Systems (Minneapolis, MN) respectively. Annexin V-PI kit was purchased from Beckman Coulter, Fullerton, CA. The PP-1 assay kit and the luciferase assay kit were purchased from New England Biolabs (Beverley, MA) and Promega (Madison, WI) respectively. Ly6E-Luciferase-Stat3 plasmid was a kind gift from Dr. Prahlad Ram (Dept. of Systems Biology, M.D. Anderson Cancer Center, and Houston, TX, USA).

### MTT Assay

The growth inhibitory activity of avicin D was measured by the MTT [3-(4, 5-dimethylthiazol-2-yl)-2, 5-diphenyl tetrazolium bromide] reduction assay as described earlier [5]. There have been reports of interference by chemotherapeutic agents in the MTT assay [64]. Any interaction between avicin D and MTT that could interfere with the assay was ruled out by evaluating various concentrations of avicin D incubated with medium alone (no cells) by the MTT assay.

### Annexin V-FITC Binding Assay

Induction of apoptosis was studied by annexin V-FITC binding assay using a kit from Beckman Coulter. Treatment and staining of cells was done as per the kit instructions. Briefly, U266 (1×10^6^) were treated with 1 µM of avicin D for 0–72 hrs at 37°C. After washing the cells in cold PBS, they were resuspended in binding buffer. Annexin V-FITC conjugate was added (1 µg/ml) and incubated for 15 min at room temperature in the dark. Cells were then stained with propidium iodide (5 µg/ml) and analyzed by flow cytometry [65].

### Treatment of cells and Western blot analysis

Cells were serum starved prior to treatment with avicins. In case of Stat3 induction by IL-6, cells were treated with 10 ng/ml of IL-6 for 30 min. Whole cell lysates were prepared from untreated and treated cells. Cellular proteins (50–100 µg) were resolved on an SDS/10% polyacrylamide gel. Levels of different proteins were analyzed by Western blot analysis using the respective antibodies. Protein bands were detected by chemiluminescence (ECL, Amersham Pharmacia) and quantitated using the Image J software.

### Immunofluorescent Staining

Serum starved U266 **c**ells were either untreated or treated with avicin D (1 µM) for 16 hrs. Cells were next exposed to IL-6 (10 ng/ml) for 30 min. After treatment, cells were washed twice with PBS, and fixed with 4% paraformaldehyde. The cells were permeabilized using 1% Triton X-100 and 0.5% NP40. After blocking the cells with 1% BSA, they were incubated with primary antibody (2 hrs) followed by secondary (Rhodamine) antibody (1 hr). Cells were then stained with 4′, 6-diamidino-2-phenylindole (DAPI) to permit visualization of nuclear DNA. The immunofluorescence was visualized by using a Leica DM LB fluorescence microscope. Images were captured with an automatic imaging system.

### Transfection and Assay of Luciferase activity

U266 cells were transfected with Ly6E-luciferase construct (4 µg) and β-galactosidase (β-gal) construct (2 µg) using the Amaxa transfection system (Amaxa, Cologne, Germany). 24 hrs post transfection, cells were washed and transferred into RPMI 1640 containing 1% FBS. Cells were then treated with avicin D for 16 hrs, followed by a 30 min exposure to IL-6 (10 ng/ml). Luciferase activity was measured by using the luciferase assay kit according to the manufacturer's protocol. Data were normalized for the β-gal activity, which was measured using the β-gal assay system from Promega.

### Assay for serine/threonine phosphatase activity

Serum starved U266 cells were treated with avicin D. Cells were lysed in lysis buffer containing 20 mM Tris HCl, 150 mM NaCl, and 1% NP-40. Protein phosphatase 1 (PP1) was immunoprecipitated from 600 µg of cellular protein using PP1 (FL-18) antibody. Using P^32^ labeled myelin basic protein as a substrate, the phosphatase activity of the immunoprecipitated PP1 was measured with the aid of the protein serine/threonine phosphatase (PSP) assay system. Labeling of the substrate and assay were performed as per the manufacturer's instructions.

### Ethics Statement

Mice were housed and cared for in accordance with the Institute of Laboratory Animal Research (ILAR) Commission on Life Sciences, National Research Council document “Guide for the care and use of laboratory animals”. The animal study protocol was reviewed by the Institutional Animal Care and use Committee at the University of Texas Health Science Center at San Antonio.

### DMBA/Avicin treatment of mice

Skin carcinogenesis was induced using DMBA as described earlier [9]. Seven-week old SENCAR mice from National Cancer Institute (Fredrick, MD) were used for the study. 100 nmol of 7, 12-Dimethylbenz[a]anthracene (DMBA) in 0.2 ml acetone was applied to the shaved dorsal areas of the mice, twice a week for 8 weeks. Avicin solutions (1 mg in 0.2 ml acetone) were applied to the shaved area of the experimental animals 15 min before DMBA application, twice a week for 8 weeks. Assessment of the performance of animals and tumor progression has been described earlier [9].

### Immunohistochemistry

Skin sections prepared by standard procedures described earlier [9], were incubated with primary antibodies against VEGF and CD31. The murine skin sections were incubated overnight with the primary antibody at 4°C followed by incubation with the appropriate biotinylated secondary antibodies (Vector laboratories Inc., Burlingame, CA, USA) for 2 h. Slides were then stained with diaminobenzidine-chromogen and counterstained with aqueous hematoxylin.

### Statistical analysis

Each experiment or assay was performed at least three times in triplicate and representative examples are shown. Data are reported as means±SD. Statistical significance between and treated samples was calculated using the Student's t-test. P<0.05 were considered significant. For the animal studies, one-way ANNOVA was performed on the measured parameters to determine the significance of changes between the treated and control groups. Again p<0.05 were considered significant.

## Supporting Information

Figure S1 Chemical nature of avicins. (A) HPLC profile of avicins. (B) Chemical structure of avicin D and avicin G.
(0.30 MB TIF)
