# Supplementary figures and images for: Avicin D: A Protein Reactive Plant Isoprenoid Dephosphorylates Stat 3 by Regulating Both Kinase and Phosphatase Activities
Source: PLoS One. 2009 May 18;4(5):e5578. doi: 10.1371/journal.pone.0005578 (PMC2680980; doi:10.1371/journal.pone.0005578)

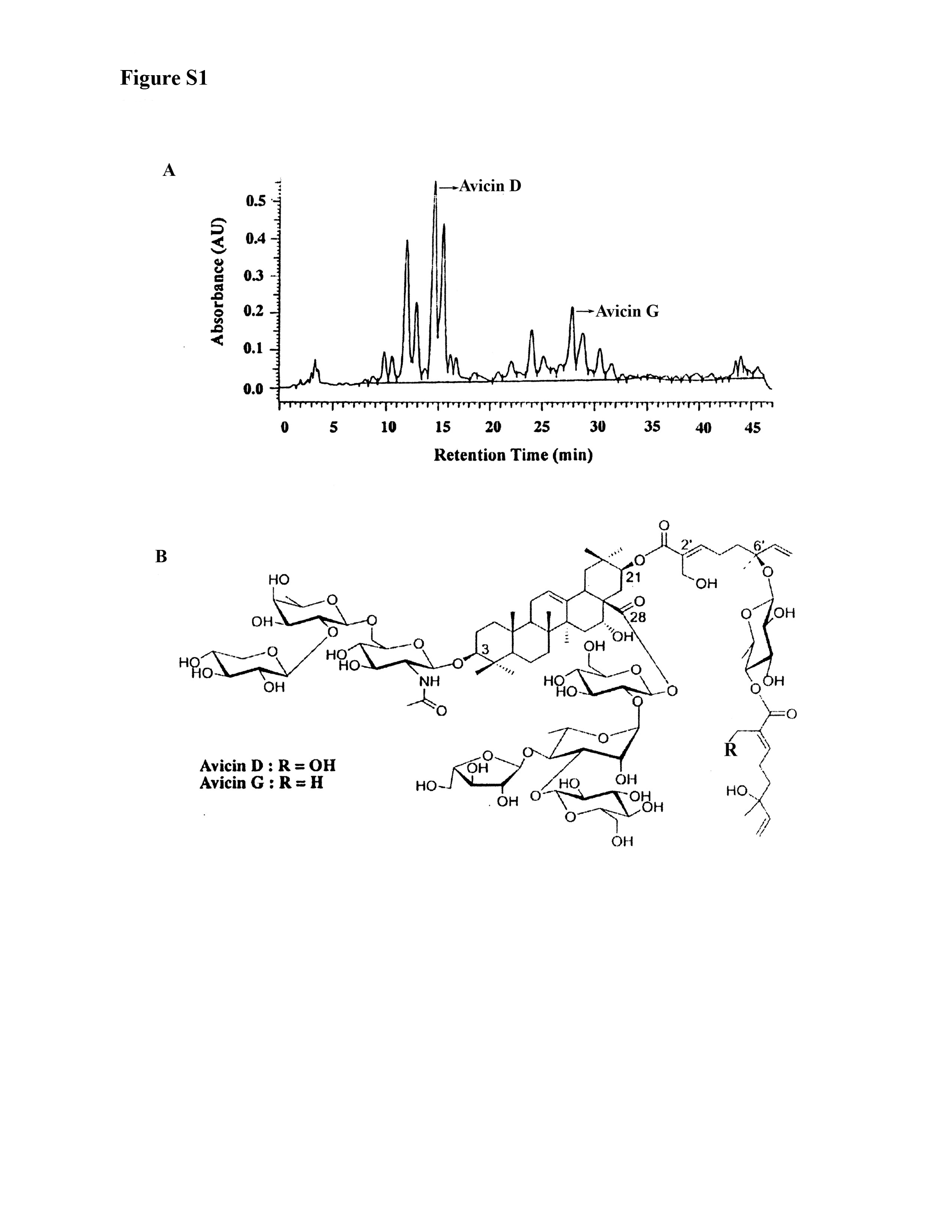

Supplement: Figure S1 — Chemical nature of avicins. (A) HPLC profile of avicins. (B) Chemical structure of avicin D and avicin G. (0.30 MB TIF) [file pone.0005578.s001.tif]
